# A Uniform Certificate for Hospital Nurses

**Published:** 1908-04-18

**Authors:** 


### Editor's Letter-Box.

**Our correspondents are reminded that prolixity is a great bar to publication, and that brevity of style and conciseness of statement greatly facilitate early insertion.**

### A UNIFORM CERTIFICATE FOR HOSPITAL NURSES.

Sir,—I am glad that you are once more urging the necessity of a uniform certificate. Do not you think, if the nurse-training schools would only support the examination of the R.B.N.A., then we should get our nurses to go in for this as a final examination, in addition to the one they already go in for in connection with their hospital certificate, and by this means get a uniform certificate?

I was asked, though not a member of the Royal British Nurses' Association, but as matron to one of the large provincial hospitals, to act as one of the examiners for the diploma. I went, and we certainly gave the nurses a very severe test in practical nursing as well as the written paper.

Would it not be well if the matrons of our large hospitals would encourage this and offer themselves as examiners? If you have any reasons against it, I should be glad to know them. Thanking you in anticipation, Matron.

